# CD38-targeted therapies in kidney disease: evidence maturity, disease-stage dependence, and biomarker-guided retreatment

**DOI:** 10.3389/fmed.2026.1972506

**Published:** 2026-10-14

**Authors:** Peidong Feng, Xiaoyong Yu

**Affiliations:** 1 First School of Clinical Medicine, Shaanxi University of Chinese Medicine, Xianyang, Shaanxi, China; 2 Department of Nephrology, Shaanxi Provincial Hospital of Traditional Chinese Medicine, Xi'an, Shaanxi, China

**Keywords:** antibody-mediated rejection, biomarker-guided retreatment, CD38, IgA nephropathy, lupus nephritis, membranous nephropathy, plasma cells

## Abstract

CD38 targeting can reach antibody-secreting cells that persist after B-cell depletion, along with selected natural killer-cell and other immune-cell subsets. This narrative review examines CD38-targeted antibodies in immune-mediated and monoclonal immunoglobulin-related kidney diseases and kidney transplantation. It asks what clinical studies establish, how disease stage may affect response durability, and whether biomarkers can guide retreatment. Evidence differs by agent, regimen, and outcome. The most mature randomized evidence with kidney-organ outcomes comes from daratumumab-based combination therapy in immunoglobulin light-chain (AL) amyloidosis, a plasma-cell disorder. In other kidney and transplant indications, early-phase randomized trials and uncontrolled studies show immunologic, tissue, or proteinuria responses, but long-term kidney or graft benefit remains unproven. Disease duration, previous treatment, and residual tissue injury may help explain variable responses, although treatment selection by disease stage has not been validated. Biomarkers measure distinct aspects of response and can support monitoring; changes alone do not establish pathogenic-cell depletion or justify retreatment. Biomarker-guided dosing was feasible in a selected transplant cohort and requires comparative validation. Clinical interpretation must remain specific to the studied antibody and regimen. Research priorities include trials with kidney and graft outcomes, prospective patient selection, comparisons of treatment strategies, validation of biomarker-guided retreatment, and cumulative safety during repeated therapy.

## 1 Introduction

Immune-mediated kidney diseases can remain difficult to control when mature, CD20-negative plasma cells continue to produce pathogenic immunoglobulins after conventional immunosuppression or B-cell depletion. CD38 is highly expressed on plasmablasts and plasma cells and is also present on selected natural killer-cell, T-cell, and myeloid subsets. Targeting CD38 therefore reaches a residual effector compartment that can sustain donor-specific alloimmunity, autoantibody production, immune-complex formation, and nephrotoxic monoclonal immunoglobulin secretion (1–4).

Previous reviews have described CD38 biology, applications across kidney diseases, and the therapeutic rationale in antibody-mediated rejection; the Kidney Disease: Improving Global Outcomes (KDIGO) Controversies Conference report placed B-cell and plasma-cell targeting within the broader management of immune-mediated kidney disease (5–8). Building on this work and subsequent clinical reports, this review examines how heterogeneous responses should inform expectations about durable organ benefit, patient selection, and repeat treatment. The unresolved clinical problem is how to connect the findings supported by each study design and endpoint with disease stage and residual injury, and how to distinguish useful biomarker monitoring from evidence that acting on a biomarker improves outcomes.

This narrative review critically appraises CD38-targeted antibodies in immune-mediated and monoclonal immunoglobulin-related kidney diseases and related transplant applications. It addresses three linked questions. What conclusions can the clinical evidence support for each antibody and indication? How might disease stage, prior treatment, and residual tissue injury influence response durability? Which biomarkers support response assessment, and what evidence is needed to use them to guide retreatment? The clinical sections address the first question, and the Discussion integrates disease-stage dependence and biomarker-guided retreatment across indications.

## 2 Methods

For this narrative review, focused, non-systematic searches were performed in PubMed/MEDLINE on 17 August 2026. Search concepts combined CD38, daratumumab, felzartamab, isatuximab, and mezagitamab with antibody-mediated rejection, kidney transplantation, desensitization, immunoglobulin A nephropathy, primary membranous nephropathy, lupus nephritis, anti-neutrophil cytoplasmic antibody-associated vasculitis, monoclonal gammopathy of renal significance, proliferative glomerulonephritis with monoclonal immunoglobulin deposits (PGNMID), light-chain deposition disease, and immunoglobulin light-chain (AL) amyloidosis. A separate mechanistic search combined CD38 with plasma cells, plasmablasts, natural killer cells, kidney, renal, and transplantation terms. No language restriction was applied at the query stage. To improve reproducibility, the clinical and mechanistic queries were specified and rerun on 10 September 2026 using a publication-date cutoff of 17 August 2026 and no substantive lower date limit. Supplementary Methods S1 provides the complete queries, field tags, date limits, and retrieval counts.

Human clinical studies were prioritized when they reported kidney, immunologic, histologic, molecular, or transplant-access outcomes. Original randomized trials, prospective cohorts, registries, and case series formed the basis of efficacy and safety statements. Reviews and guidelines were used for biological background and clinical context. Conference abstracts and preprints were labeled as preliminary reports when a full peer-reviewed report was unavailable, with publication status recorded separately from study design. The revision-stage search was deduplicated by PubMed identifier (PMID), with the digital object identifier (DOI) used to check record identity. Overlapping clinical reports in the evidence tables were linked using trial identifiers, recruitment setting, authors, sample characteristics, and treatment schedules where available. For overlapping cohorts, the most complete or longest-follow-up report informed the core evidence matrix; earlier reports or extensions were retained for distinct mechanistic, endpoint, or retreatment information and were not counted as independent replication. Studies confined to hematologic malignancy without a kidney or mechanistic contribution were excluded from the kidney-efficacy synthesis.

ClinicalTrials.gov was searched and checked on 17 August 2026 to verify trial status, enrollment, design, primary endpoints, and completion dates. The two additional transplant trials requested during review were checked on 10 September 2026 and are dated separately in Table 1. Bibliographic metadata were checked against PubMed and Crossref. For each clinical report, the indication, agent, design, sample size, comparator, regimen, principal outcome, follow-up, publication status, and major limitation were extracted. Registrations without peer-reviewed efficacy results and preclinical studies were considered separately from published human clinical evidence.

**Table 1:** Ongoing or recently completed kidney trials without peer-reviewed efficacy results. The five original entries retain their 17 August 2026 registry snapshot; TRANSPIRE and the TRANSCEND/TRANSPIRE extension were checked on 10 September 2026. Planned dates and enrollment may change.

Trial and indication	Agent	Design and planned enrollment	Registry status	Principal outcome	Completion	Reference
TRANSCEND; late AMR	Felzartamab	Phase 3, randomized, quadruple-masked; *n* = 132	Active, not recruiting	Biopsy-defined AMR resolution at week 24	Primary completion December 2026	NCT06685757 (9)
PREVAIL; IgAN	Felzartamab	Phase 3, randomized, quadruple-masked; *n* = 454	Recruiting	Proteinuria and longer-term kidney-function outcomes	Primary completion May 2027	NCT06935357 (10)
Phase 3 IgAN program	Mezagitamab	Phase 3, randomized, quadruple-masked; *n* = 347	Recruiting	Proteinuria at week 36 with eGFR follow-up	Primary completion September 2028	NCT06963827 (11)
PROMINENT; pMN	Felzartamab	Phase 3, randomized, open label; *n* = 180	Recruiting	Complete proteinuria remission at week 104	Primary completion January 2030	NCT06962800 (12)
Phase 1b lupus nephritis	Felzartamab	Phase 1, single-group; *n* = 14	Completed; results not yet incorporated as published efficacy	Safety and kidney-response measures	Completed July 2026	NCT06064929 (13)
TRANSPIRE; late DSA-negative MVI	Felzartamab	Phase 2; randomized, quadruple-masked Part A; open-label Part B; *n* = 81	Recruiting	Biopsy-proven histologic resolution at week 24	Primary completion February 2028	NCT07219043 (14)
TRANSCEND/TRANSPIRE LTE; AMR or MVI	Felzartamab	Phase 3; open-label, single-group extension; *n* = 201	Enrolling by invitation	Long-term safety through weeks 200–204	Primary completion May 2031	NCT07444489 (15)

AMR, antibody-mediated rejection; DSA, donor-specific antibody; eGFR, estimated glomerular filtration rate; IgAN, immunoglobulin A nephropathy; LTE, long-term extension; MVI, microvascular inflammation; pMN, primary membranous nephropathy.

Evidence was appraised descriptively without an ordinal hierarchy. Developmental maturity refers to trial phase, and publication maturity to the availability of complete reports; methodological quality concerns susceptibility to bias; certainty concerns confidence in the evidence for a specific outcome, as addressed by the Grading of Recommendations Assessment, Development and Evaluation (GRADE) approach (16). We did not undertake a formal GRADE assessment. Study design and publication status were recorded separately, so preliminary publication did not change a report's stated design. Sample size qualified precision; comparators and co-interventions qualified attribution; endpoint type, primary or secondary status, and follow-up limited the conclusions supported. A short trial with a biomarker endpoint cannot establish a survival benefit, and findings were not assumed to transfer across antibodies or indications. No protocol was registered, duplicate independent screening was not performed, and no formal risk-of-bias instrument or meta-analysis was applied.

## 3 Mechanistic basis and agent-level differences

### 3.1 CD38 biology relevant to kidney disease

CD38 is encoded on chromosome 4 and forms a 45-kDa transmembrane protein with a short cytoplasmic tail, a single membrane-spanning region, and a large extracellular catalytic domain. Its enzymatic activity consumes extracellular nicotinamide adenine dinucleotide and generates adenosine diphosphate ribose, cyclic adenosine diphosphate ribose, and nicotinic acid adenine dinucleotide phosphate. These metabolites regulate calcium mobilization, mitochondrial activity, cell activation, and survival. CD38 also participates in the CD38-CD203a-CD73 pathway, which converts extracellular nucleotide substrates into immunoregulatory adenosine. Its receptor and ectoenzymatic functions connect cell-surface phenotype with tissue metabolism (1, 4).

CD38 expression changes during lymphocyte differentiation. Naive B cells express modest levels, germinal-center cells and activated B cells increase expression, memory B cells generally remain lower, and plasmablasts and plasma cells display the highest density. This pattern makes CD38 a functional marker of terminal antibody-secreting differentiation. Long-lived plasma cells in bone marrow and inflamed tissues can maintain antibody production for years, survive the removal of upstream B cells, and preserve autoreactive or alloreactive humoral memory. In kidney disease, these cells sustain donor-specific human leukocyte antigen (HLA) antibodies, anti-phospholipase A2 receptor (PLA2R) or anti-thrombospondin type-1 domain-containing 7A (THSD7A) autoantibodies, anti-double-stranded DNA antibodies, and nephrotoxic monoclonal immunoglobulins. Plasma-cell targeting aims to reduce the cellular source of these antibodies; complement and Fc-receptor-bearing cells mediate downstream effector injury.

Natural killer cells complicate the mechanism because activated and cytotoxic subsets also express abundant CD38. Anti-CD38 antibodies reduce these cells through fratricide and Fc-dependent pathways. Depletion of CD38-positive natural killer cells may reduce antibody-dependent cellular cytotoxic capacity and may also reduce the natural killer-cell contribution to microvascular inflammation. Their transcripts and endothelial injury programs are prominent in antibody-mediated rejection. Effects on selected natural killer cells may contribute to the histological changes observed with felzartamab even when donor-specific antibody remains detectable; the relative contribution of plasma-cell depletion remains uncertain (17, 18).

Expression outside plasma and natural killer cells is more variable. Activated CD4-positive and CD8-positive T cells, regulatory T cells, monocytes, dendritic cells, and myeloid-derived suppressor cells all contain CD38-positive subsets. Daratumumab can remove immunosuppressive CD38-positive populations and expand effector T-cell clonality in myeloma; autoimmune studies likewise show changes in activated T cells and plasmablasts. Several mechanisms may therefore contribute in kidney disease. Reduced antibody production and altered natural killer-cell activity are plausible contributors in alloimmune disease; changes in autoantibodies and interferon-linked activity may contribute in systemic autoimmunity. In plasma-cell clonal disease, suppression of the pathogenic immunoglobulin-producing clone is the therapeutic objective. Their relative contributions vary by disease and antibody and are not established by kidney response alone (2, 3, 19).

Four Fc-dependent mechanisms contribute to depletion: antibody-dependent cellular cytotoxicity, antibody-dependent cellular phagocytosis, complement-dependent cytotoxicity, and direct apoptosis after crosslinking. Their relative contribution varies by antibody, target density, complement-regulatory proteins, Fc-receptor engagement, prior treatment, and tissue accessibility (20–22). Table 2 summarizes the principal CD38-expressing compartments, and Figure 1 maps these compartments to kidney injury pathways.

**Table 2:** Principal CD38-expressing cell populations and their relevance to kidney disease. Supporting sources: (1–4, 17, 18).

Cell population	Relative CD38 expression	Principal function	Kidney pathogenic relevance	Expected effect of CD38 targeting
Plasmablasts	Very high	Rapid antibody secretion and expansion during active immune responses	Active autoantibody and alloantibody production	Rapid suppression of newly generated pathogenic antibody
Long-lived plasma cells	Very high	Sustained antibody secretion independent of CD20-positive B cells	Persistent DSA, anti-PLA2R/THSD7A, anti-dsDNA, IgA, and monoclonal immunoglobulin production	Potential reduction of persistent antibody secretion; tissue depletion may be incomplete
Natural killer cells	High	Cytotoxicity, Fc-receptor signaling, endothelial interaction	Molecular and cellular contribution to microvascular inflammation	Potential reduction of NK-mediated injury and ADCC capacity; duration and immune-surveillance effects require study
Activated T cells	Intermediate to high	Cytokine production, cytotoxicity, help for B-cell differentiation	Maintenance of alloimmune and autoimmune activation	Agent- and setting-dependent changes in activated CD38-positive subsets
Regulatory T cells and regulatory B cells	Variable	Control of immune activation	Modulation of tolerance and inflammatory thresholds	Context-dependent redistribution during immune reconstitution
Monocytes and dendritic cells	Variable	Antigen presentation, phagocytosis, cytokine release	Immune-complex handling and inflammatory amplification	Altered Fc-mediated clearance and inflammatory signaling
Erythrocytes	Low	Blood-group serologic substrate	No pathogenic kidney role; source of compatibility-test interference	Persistent positive indirect antiglobulin testing during and after treatment

ADCC, antibody-dependent cellular cytotoxicity; DSA, donor-specific antibody; dsDNA, double-stranded DNA; IgA, immunoglobulin A; NK, natural killer; PLA2R, phospholipase A2 receptor; THSD7A, thrombospondin type-1 domain-containing 7A.

**Figure 1:**
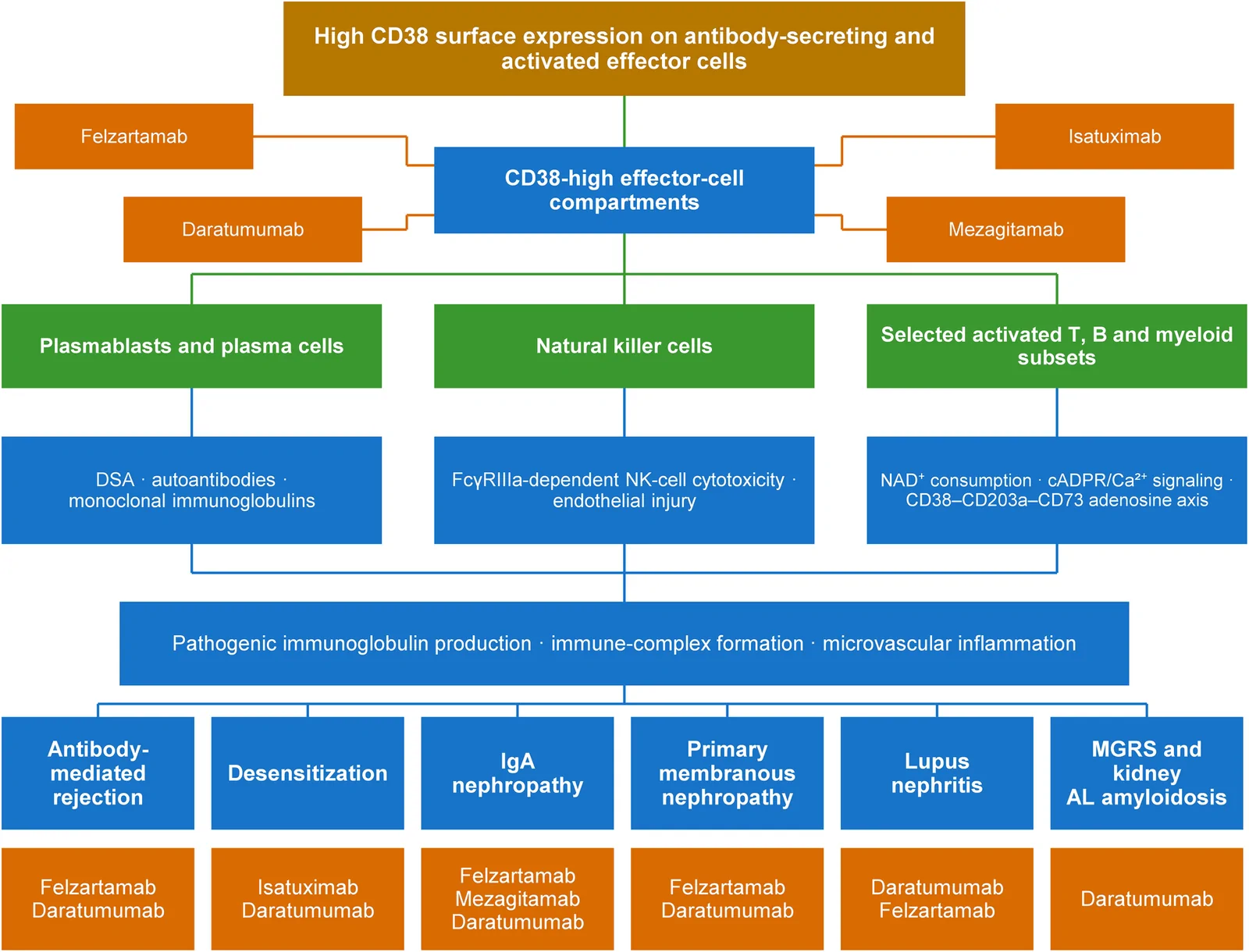
CD38-high effector-cell compartments and therapeutic intervention points in kidney disease. Anti-CD38 antibodies deplete CD38-high plasma cells and selected effector subsets, suppressing pathogenic immunoglobulin production and downstream injury pathways. AL amyloidosis, immunoglobulin light-chain amyloidosis; cADPR, cyclic adenosine diphosphate ribose; DSA, donor-specific antibody; Fc*γ*RIIIa, Fc gamma receptor IIIa; IgA, immunoglobulin A; MGRS, monoclonal gammopathy of renal significance; NAD^+^, nicotinamide adenine dinucleotide; NK, natural killer. Conceptual synthesis based on (1–4, 17–22). The pathways indicate potential cellular contributions rather than proven causal mechanisms in every indication; agent-indication links include differing stages of clinical development and do not imply equivalent efficacy.

### 3.2 Differences among CD38-targeted antibodies

Shared target expression does not make the available antibodies interchangeable. Felzartamab is dominated by antibody-dependent cellular cytotoxicity and phagocytosis with limited complement-dependent cytotoxicity; daratumumab combines complement, cellular cytotoxicity, phagocytosis, apoptosis, and immune modulation; isatuximab binds a distinct epitope and also inhibits CD38 enzymatic activity; and mezagitamab is a fully human cytolytic antibody developed for subcutaneous use in autoimmune disease (20–22). Formulation, dosing intensity, established hematology infrastructure, and the depth of kidney-specific evidence therefore influence where each agent can be implemented.

Felzartamab has the broadest kidney-specific clinical development program, whereas daratumumab has the greatest cumulative clinical exposure and randomized phase 3 evidence for combination therapy in a plasma-cell disorder with kidney-organ endpoints. Isatuximab's clearest kidney-specific evidence concerns transplant desensitization. Mezagitamab uses a subcutaneous regimen designed for repeated finite courses. Table 3 compares these pharmacologic and development features without treating ongoing programs as efficacy evidence. Shared targets and plausible mechanisms do not establish equivalent benefit. Clinical evidence therefore needs to be assessed for each antibody and indication.

**Table 3:** Pharmacologic and development differences among CD38-targeted agents used or studied in kidney disease.

Agent	Antibody and principal mechanisms	Administration	Leading kidney indications	Published kidney evidence or established use	References
Felzartamab (MOR202/BIIB148)	Fully human IgG1 lambda; ADCC and ADCP dominant; limited CDC	Intravenous, weight-based	Late AMR, IgAN, pMN, LN	Kidney-specific randomized phase 2 and prospective evidence; phase 3 programs listed separately	(20, 23–26)
Daratumumab	Fully human IgG1 kappa; CDC, ADCC, ADCP, apoptosis, immune modulation	Intravenous or fixed-dose subcutaneous	AL amyloidosis, PGNMID, light-chain deposition disease, refractory LN, AMR	Approved in plasma-cell disorders; randomized phase 3 organ-response evidence in AL amyloidosis	(20, 27–34)
Isatuximab	Chimeric IgG1 kappa; ADCC, ADCP, apoptosis, enzymatic inhibition	Intravenous; subcutaneous formulation used in hematology	Pre-transplant desensitization	Prospective phase 1/2 kidney evidence	(21, 35, 36)
Mezagitamab (TAK-079)	Fully human IgG1 lambda cytolytic antibody; plasma-cell and NK-cell depletion	Subcutaneous	IgAN, systemic lupus erythematosus	Phase 1b evidence; phase 3 IgAN development listed separately	(11, 22, 37–40)

ADCC, antibody-dependent cellular cytotoxicity; ADCP, antibody-dependent cellular phagocytosis; AL, immunoglobulin light-chain; AMR, antibody-mediated rejection; CDC, complement-dependent cytotoxicity; IgAN, immunoglobulin A nephropathy; IgG1, immunoglobulin G1; LN, lupus nephritis; NK, natural killer; PGNMID, proliferative glomerulonephritis with monoclonal immunoglobulin deposits; pMN, primary membranous nephropathy.

## 4 Clinical evidence across kidney diseases

Tables 4, 5 summarize the core clinical reports, including their comparators, outcomes, publication status, and limitations. The following sections distinguish observed activity from evidence of durable kidney or graft benefit.

**Table 4:** Core published clinical evidence for CD38-targeted therapy in kidney transplantation and immune-mediated glomerular disease. Study design, publication status, response domains, and limitations are described separately.

Indication	Agent and reference	Design and sample	Comparator	Regimen and follow-up	Principal finding	Main limitations
Late AMR	Felzartamab (23)	Randomized, double-blind phase 2; *n* = 22	Placebo	Nine IV doses over 24 weeks; biopsy at weeks 24 and 52	Morphologic AMR resolution 82% vs 20%; lower MVI, molecular AMR score, and dd-cfDNA; recurrence in 3/9 initial histological responders by week 52	Small trial; safety primary endpoint; exploratory functional findings; no established graft-survival benefit; industry funded
Persistent or recurrent AMR	Felzartamab (41)	Biomarker-guided open-label extension; *n* = 11	None	Additional 12 months; fixed dosing followed by dd-cfDNA-guided dosing	Median MVI 2 to 0; 7/11 reached MVI 0; median two doses in biomarker-guided phase	No concurrent control; selected cohort; exploratory dosing thresholds; persistent AMR despite low dd-cfDNA possible; graft benefit unknown
Post-transplant MVI	Daratumumab (27)	Multicenter retrospective registry; *n* = 70	Pre/post comparison	Variable real-world regimens	eGFR slope changed from −1.6 to +0.3 mL/min/1.73 m²/month; albuminuria and dd-cfDNA fell	Confounding by indication and co-interventions; heterogeneous dosing; six graft losses
Transplant desensitization	Isatuximab (35)	Open-label phase 1/2; *n* = 23	None	Weekly for 4 weeks then every 2 weeks for 8 weeks (8 doses in total); median follow-up 68 weeks	Protocol-defined desensitization response 82–83%; target cPRA reduction in 9/23; six offers and four accepted transplants	Laboratory response translated into transplantation for few participants; no comparator
IgAN	Felzartamab (24, 42)	Phase 2a; 48 randomized plus six Japanese open-label participants	Placebo plus three dose groups	Two, five, or nine doses; randomized follow-up to 24 months; Japanese cohort to 12 months	Randomized nine-dose UPCR change −29.5% at month 9 and −44.5% at month 24; Japanese cohort −44.8% at month 9; eGFR stable	Small dose groups; proteinuria surrogate; kidney-failure benefit unproven
IgAN	Mezagitamab (37–39)	Open-label phase 1b; *n* = 17	None	Subcutaneous treatment; reported follow-up to 96 weeks	Lower IgA, Gd-IgA1, proteinuria, and hematuria with stable eGFR	Results remain abstract-only; small uncontrolled cohort; detailed peer-reviewed methods unavailable
Anti-PLA2R-positive pMN	Felzartamab (25)	Open-label phase 1b/2a; *n* = 31; 26 efficacy-evaluable	None	Nine IV doses over 24 weeks; median follow-up 364 days	Best immunologic response 76.9%; proteinuria partial remission 34.6% at study end	Primary endpoint was safety; no comparator; subgroup differences and incomplete efficacy set
Rituximab-resistant pMN	Felzartamab (43)	Prospective single-arm cohort; *n* = 10	None	Nine-dose course; 24-month follow-up	Primary proteinuria endpoint not improved; anti-PLA2R decline was transient and incomplete	Small, single-center cohort; longstanding resistant disease; clinical differences limit cross-study comparison; tissue depletion not measured
Refractory LN	Daratumumab (28)	Kidney-biopsied case series; *n* = 6	None	Extended IV regimen; 12-month assessment	Five renal responses; responders’ means at 12 months: proteinuria 5.6 to 0.8 g/day; creatinine 2.3 to 1.5 mg/dL	Case series; no comparator; concomitant treatment and selection effects

AMR, antibody-mediated rejection; cPRA, calculated panel-reactive antibody; dd-cfDNA, donor-derived cell-free DNA; eGFR, estimated glomerular filtration rate; Gd-IgA1, galactose-deficient immunoglobulin A1; IgA, immunoglobulin A; IgAN, immunoglobulin A nephropathy; IV, intravenous; LN, lupus nephritis; MVI, microvascular inflammation; PLA2R, phospholipase A2 receptor; pMN, primary membranous nephropathy; UPCR, urine protein-to-creatinine ratio.

**Table 5:** Core published clinical evidence for CD38-targeted therapy in monoclonal immunoglobulin-related and other kidney diseases. Study design, publication status, response domains, and limitations are described separately.

Indication	Agent and reference	Design and sample	Comparator	Regimen and follow-up	Principal finding	Main limitations
Active SLE, including nephritis	Daratumumab (29)	Single-arm phase 2; *n* = 10; six with active nephritis	None	Eight weekly subcutaneous doses; primary assessment at week 12	All met systemic SRI-4 response; anti-dsDNA fell; hypogammaglobulinemia requiring IVIG in 5/10	Systemic rather than kidney primary endpoint; very small cohort; short follow-up
PGNMID	Daratumumab (30)	Open-label phase 2; 11 PGNMID plus one C3G; 10 PGNMID evaluable	None	Sixteen IV doses; 12-month follow-up	All ten evaluable PGNMID participants had renal responses; median proteinuria 4,346 to 1,264 mg/day; two improved after daratumumab restart	No comparator; one non-completer; small and biologically selected population
Newly diagnosed AL amyloidosis	Daratumumab plus VCd (32, 33)	Randomized phase 3; *n* = 388	VCd alone	Six cycles followed by daratumumab maintenance; final median follow-up 61.4 months	Hematologic complete response and renal response improved; final analysis showed better organ-deterioration-free and overall survival	Assesses daratumumab added to VCd; monotherapy efficacy and class effects remain untested; selected trial population
High-risk IgAN	Daratumumab (44)	Case series; *n* = 4	None	Variable rescue treatment; median follow-up 13.5 months	Proteinuria and hematuria improved with stable or improved kidney function	Very small series; uncontrolled; high risk of selection and publication bias

AL, immunoglobulin light-chain; C3G, C3 glomerulopathy; dsDNA, double-stranded DNA; IgAN, immunoglobulin A nephropathy; IV, intravenous; IVIG, intravenous immunoglobulin; PGNMID, proliferative glomerulonephritis with monoclonal immunoglobulin deposits; SLE, systemic lupus erythematosus; SRI-4, Systemic Lupus Erythematosus Responder Index-4; VCd, bortezomib, cyclophosphamide, and dexamethasone.

### 4.1 Kidney transplantation

Felzartamab has been evaluated in a randomized phase 2 trial of late antibody-mediated rejection (AMR). Donor-specific antibodies bind allograft endothelium, activate Fc-dependent and complement-dependent injury, and recruit natural killer cells and macrophages. Banff assessment integrates histology, evidence of antibody-endothelium interaction, donor-specific antibodies, and appropriately validated molecular diagnostics. Late AMR describes timing; the randomized felzartamab trial enrolled recipients at least 180 days after transplantation. Chronic active AMR is a pathological category requiring chronic structural injury with evidence of current antibody-mediated activity, irrespective of time after transplantation. Microvascular inflammation (MVI), quantified by glomerulitis plus peritubular capillaritis scores, is a lesion rather than a synonym for AMR. The Banff phenotype of MVI with negative donor-specific antibodies and negative C4d is distinguished from definite AMR. Study-specific definitions are retained below (23, 45, 46).

Plasma cells sit upstream of this process. Current transplant regimens and rescue therapies suppress T cells, remove circulating antibody, inhibit complement, or deplete CD20-positive B cells. Plasma exchange and intravenous immunoglobulin can reduce antibody burden transiently; rituximab prevents generation from CD20-positive precursors; proteasome inhibitors can deplete plasma cells with variable tolerability. Persistent long-lived plasma cells remain a reservoir for continued donor-specific antibody production. This reservoir contributes to the poor durability of available treatment and to the absence of a uniformly effective standard for late or chronic active AMR (47, 48).

Twenty-two kidney-transplant recipients with late AMR received nine intravenous doses of felzartamab 16 mg/kg or placebo over 24 weeks. At week 24, AMR had resolved morphologically in 9 of 11 felzartamab-treated participants and 2 of 10 placebo-treated participants. Median microvascular inflammation was 0 with felzartamab and 2.5 with placebo; complete absence of microvascular inflammation occurred in 64% and 10%, respectively. Molecular AMR activity and donor-derived cell-free DNA also improved. The exploratory annual estimated glomerular filtration rate slope was −0.39 mL/min/1.73 m^2^ with felzartamab and −4.53 mL/min/1.73 m^2^ with placebo. The trial primarily assessed safety; the histological, molecular, injury-biomarker, and exploratory functional findings do not establish improved long-term graft survival (23, 49).

Molecular biopsy analysis showed suppression of interferon-*γ*-inducible and natural killer-cell-associated AMR transcripts, with little effect on stage-associated endothelial genes. Donor-specific antibody concentrations did not consistently fall with the histological and molecular changes. Together with peripheral natural killer-cell depletion, these findings support a contribution from downstream effector pathways, although the relative effects on natural killer cells and plasma cells remain unresolved. Donor-derived cell-free DNA and molecular AMR scores provide complementary measures of injury and rejection activity; comparative accuracy for directing treatment remains unproven (18, 50).

Histological AMR activity could recur after finite treatment. Three of nine initial histologic responders relapsed after the nine-dose course. In the biomarker-guided extension, 11 participants with persistent or recurrent AMR received six months of fixed felzartamab dosing followed by six months of donor-derived cell-free DNA-guided dosing. Median microvascular inflammation fell from 2 to 0 at week 52, 7 of 11 reached a score of 0, and molecular AMR probability declined from 0.77 to 0.12. Once dosing was guided by donor-derived cell-free DNA, participants received a median of two additional doses. This selected, uncontrolled cohort demonstrates the feasibility of prospectively applying biomarker-guided dosing. It does not validate the thresholds, establish superiority over fixed dosing, or demonstrate long-term graft benefit; persistent or recurrent AMR could occur despite low donor-derived cell-free DNA (41).

The daratumumab record provides separate observational evidence. Case reports and small series described lower donor-specific antibodies, donor-derived cell-free DNA, creatinine, and microvascular inflammation in refractory AMR (51–53). A retrospective cohort used subcutaneous daratumumab 1,800 mg weekly for four doses followed by three quarterly doses and reported complete molecular resolution of AMR in 8 of 16 recipients (54). In a later multicenter registry of 70 recipients with microvascular inflammation, the monthly estimated glomerular filtration rate slope changed from −1.6 before treatment to +0.3 mL/min/1.73 m^2^ after treatment; albuminuria and donor-derived cell-free DNA also fell. Donor-specific antibody responses varied despite these improvements, supporting complementary tissue and injury-biomarker assessment without establishing a treatment-guidance rule (27).

The randomized felzartamab study provides a controlled signal in intermediate endpoints, while its 11-patient extension adds retreatment-feasibility data and the separate 70-recipient daratumumab registry adds uncontrolled outcome associations (23, 27, 41). Histology, molecular AMR activity, donor-derived cell-free DNA, kidney-function slope, and safety should remain distinct endpoints because donor-specific antibody concentrations may not track tissue response.

Highly sensitized candidates can remain on the waiting list despite kidney-allocation priority because broad and high-strength anti-HLA antibodies prevent a compatible match. Plasma exchange, intravenous immunoglobulin, anti-CD20 therapy, proteasome inhibition, and imlifidase can create temporary access to transplantation, yet antibody rebound and incomplete removal of long-lived plasma cells limit their reach. CD38 targeting directly addresses the cellular source of anti-HLA antibodies; effects on HLA-specific memory depend on the agent and the populations measured.

Isatuximab was evaluated as monotherapy in a phase 1/2 open-label study of 23 highly sensitized candidates. Participants received 10 mg/kg weekly for four weeks and then every two weeks for eight weeks (eight doses in total). A protocol-defined desensitization response occurred in 83.3% of candidates with calculated panel-reactive antibody of at least 99.90% and 81.8% of those with values from 80% to below 99.90%. Nine of 23 achieved the target reduction in calculated panel-reactive antibody, six received transplant offers, and four accepted transplantation. Plasmablasts, plasma cells, natural killer cells, and HLA-specific IgG memory B cells all declined. The intervention changed both circulating antibody and the cellular compartments capable of rebuilding the alloantibody repertoire (35).

Daratumumab has also reduced donor-specific antibodies in sensitized nonhuman primates and highly sensitized patients, although antibody rebound can follow immune reconstitution (55). The reduction window must coincide with allocation or living-donor planning, and post-transplant monitoring must then detect early rebound. The clinical value of the observed immunologic changes depends on whether they enable a suitable transplant opportunity. A laboratory response without organ access leaves the clinical objective unfinished.

### 4.2 Immune-mediated glomerular diseases

Immunoglobulin A nephropathy (IgAN) begins with production of galactose-deficient immunoglobulin A1 and antiglycan antibodies. Their circulating immune complexes deposit in the mesangium and trigger local inflammatory injury. CD38-high plasmablasts and plasma cells supply the initiating immunoglobulin and autoantibody components, whereas complement activation and intrarenal inflammation propagate the lesion after deposition. This separation makes plasma-cell depletion an upstream intervention in the multi-hit process (56, 57).

The phase 2a IGNAZ study included 48 adults randomized to two, five, or nine felzartamab infusions or placebo and a separate six-participant Japanese open-label cohort, all with biopsy-confirmed IgAN receiving stable background therapy. At month 9, least-squares mean changes in urine protein-to-creatinine ratio were −5.7% with placebo, −12.5% with two doses, −12.8% with five doses, and −29.5% with nine doses. The small Japanese open-label cohort (*n* = 6) had a −44.8% change at month 9 and was followed through month 12. In the randomized nine-dose group, the reduction was −44.5% at month 24, after treatment had ended. These proteinuria findings do not by themselves establish the depth of pathogenic-cell depletion or a long-term kidney-survival benefit (24, 42).

Proteinuria remained lower after the last felzartamab infusion, and estimated glomerular filtration rate remained stable during phase 2 follow-up. The dose gradient and persistence are consistent with an immune-modifying effect, although preservation of long-term kidney function remains unproven (24, 42).

Subcutaneous mezagitamab was associated with immunologic and proteinuria changes in a separate uncontrolled study reported in conference abstracts. In a phase 1b study, 17 participants received 600 mg weekly for eight weeks and then every two weeks for sixteen weeks. At week 48, mean immunoglobulin A fell by 52.4%, galactose-deficient immunoglobulin A1 by 57.8%, and urine protein-to-creatinine ratio by 54.1%; estimated glomerular filtration rate remained stable. At week 96, proteinuria remained approximately 55% below baseline, hematuria had resolved in 60%, and kidney function was unchanged (37–39). The randomized felzartamab findings provide a controlled proteinuria signal; the Japanese open-label cohort and abstract-only mezagitamab reports add uncontrolled observations. These studies do not establish equivalent efficacy between agents, durable depletion of pathogenic cells, or prevention of kidney failure.

Primary membranous nephropathy (pMN) is an autoantibody-mediated glomerular disease in which antibodies against podocyte antigens, most commonly PLA2R and less frequently THSD7A, form subepithelial immune deposits and activate complement. Anti-PLA2R concentration tracks immunologic activity, precedes clinical changes in proteinuria, and provides an immunologic response marker; it does not directly measure plasma-cell depletion (58, 59). Anti-CD20 therapy is an established first-line immunologic treatment in patients at risk of progression, yet long-lived CD20-negative plasma cells can maintain autoantibody production after complete B-cell depletion (60).

The phase 1b/2a M-PLACE study evaluated a planned nine-infusion intravenous felzartamab regimen (16 mg/kg over 24 weeks) in 31 anti-PLA2R-positive participants. Eighteen were newly diagnosed or relapsing and 13 had refractory disease. Among 26 participants evaluable for efficacy, 20 achieved a best immunologic response, defined as at least a 50% reduction in anti-PLA2R antibodies for partial response or a titer below 14 RU/mL for complete response. Best immunologic response occurred in 86.7% of the newly diagnosed or relapsing cohort and 63.6% of the refractory cohort. Partial proteinuria remission at study end occurred in 34.6% overall and was more common in newly diagnosed or relapsing disease. Serum albumin increased in 20 of 26 participants. Anti-PLA2R fell before albumin recovered and proteinuria improved, consistent with an immunologic response followed by a delayed kidney response; these observations do not measure depletion of the pathogenic plasma-cell compartment (25).

The prospective MONET cohort enrolled ten patients with longstanding rituximab-resistant pMN who were scheduled to receive the same nine-dose felzartamab course. Twenty-four-hour proteinuria did not fall significantly at 12 or 24 months, no patient achieved complete remission, and anti-PLA2R reductions were incomplete or transient. Three patients achieved partial remission during follow-up; two subsequently relapsed. These findings indicate limited proteinuria benefit from the tested finite regimen in this selected cohort. Peripheral immunophenotyping showed no sustained reduction in circulating plasmablast or plasma-cell proportions, but tissue compartments were not measured. Differences in baseline characteristics, prior treatment, and follow-up limit direct comparison with M-PLACE (25, 43).

Individual daratumumab reports describe immunologic and proteinuria improvement in refractory PLA2R-positive and THSD7A-positive disease. In THSD7A-positive pMN, antibodies disappeared by month 4 and proteinuria fell below 3 g/g. Relapse followed seven months after treatment withdrawal (61, 62). The antibody recurrence supports antigen-specific monitoring; it does not establish cellular repopulation or validate preemptive retreatment before nephrotic proteinuria returns.

Lupus nephritis (LN) combines autoantibody production, nucleic-acid immune complexes, complement activation, interferon signaling, and intrarenal lymphoid and myeloid inflammation. Standard induction regimens reduce inflammation and B-cell activation, while long-lived plasma cells can preserve anti-double-stranded DNA and other autoreactive antibodies. This compartment becomes especially important in relapsing disease that persists after mycophenolate, cyclophosphamide, calcineurin inhibition, belimumab, or rituximab (63).

The first daratumumab experience involved two patients with life-threatening systemic lupus erythematosus; both improved rapidly in clinical and serologic measures after depletion of nonmalignant autoreactive plasma cells (64). A subsequent study treated six patients with refractory LN. Five had a renal response by month 3, with three complete and two partial renal responses. Among the five responders, mean proteinuria declined from 5.6 to 0.8 g/day, mean serum creatinine from 2.3 to 1.5 mg/dL, and mean Systemic Lupus Erythematosus Disease Activity Index from 10.8 to 3.6 at month 12. Improvement was maintained during continued daratumumab treatment (28).

A phase 2 study then gave eight weekly subcutaneous doses of daratumumab 1,800 mg to ten patients with active systemic lupus erythematosus. All achieved a Systemic Lupus Erythematosus Responder Index-4 (SRI-4) response at week 12. Median disease activity fell from 12 to 4, anti-double-stranded DNA antibodies decreased, and the six participants with active nephritis had a median urine protein-to-creatinine ratio reduction from 649 to 302 mg/g. Rapid disease control came with a substantial humoral cost: five patients developed hypogammaglobulinemia that required intravenous immunoglobulin (29). This intensive regimen requires immunoglobulin surveillance and replacement.

Mezagitamab has reduced plasma cells, autoantibodies, and disease activity in systemic lupus erythematosus (40). These systemic mezagitamab findings do not establish kidney-specific efficacy. Separately, the daratumumab phase 2 study enrolled only ten participants, six with active nephritis, and used systemic lupus rather than kidney response as the principal efficacy frame (29).

### 4.3 Monoclonal immunoglobulin-related kidney diseases

Monoclonal gammopathy of renal significance (MGRS) comprises kidney lesions caused by a nephrotoxic monoclonal immunoglobulin from a B-cell or plasma-cell clone that does not meet conventional criteria for overt hematologic malignancy. Even a small clone can drive progressive kidney injury because toxicity depends on immunoglobulin structure and tissue interaction, not tumor burden. Once the monoclonal protein causes proliferative glomerulonephritis, deposition disease, amyloidosis, cryoglobulinemic injury, or complement dysregulation, treatment must be directed at the clone (65, 66).

PGNMID provides a rationale for plasma-cell-directed treatment even when the responsible clone is not identified. In a phase 2 study, 11 patients with PGNMID and one with C3 glomerulopathy received daratumumab. All ten evaluable PGNMID participants had a renal response at 12 months, including four complete and six partial responses, and median proteinuria fell from 4,346 to 1,264 mg/day. Three later relapsed; two had renewed renal responses when daratumumab was restarted (30). The kidney disease relapses and improvement after retreatment support daratumumab activity in selected patients, but do not directly demonstrate repopulation or depletion of a clinically occult pathogenic clone. One patient with PGNMID had marked proteinuria reduction after low-dose daratumumab given with prednisone and valsartan (67).

In a daratumumab-treated light-chain deposition disease cohort, rapid and deep hematologic responses were accompanied by stabilization or improvement of kidney function, including in patients previously exposed to proteasome inhibitors and autologous transplantation (31). In immunoglobulin light-chain amyloidosis, the kidney objective is equally direct: suppress the amyloidogenic light chain, stop continued fibril formation, and permit organ recovery. In a pretreated cohort, daratumumab produced a 77% hematologic response and a 52% renal response among patients with kidney involvement (68).

The phase 3 ANDROMEDA trial established subcutaneous daratumumab with bortezomib, cyclophosphamide, and dexamethasone as a first-line regimen for newly diagnosed systemic light-chain amyloidosis. Hematologic complete response occurred in 53.3% with daratumumab-based therapy and 18.1% with control therapy. The six-month renal response was 53.0% and 23.9%, respectively (32). Final follow-up confirmed sustained disease control and survival benefit (33). These randomized findings establish the benefit of adding daratumumab to the tested regimen in a plasma-cell disorder; they do not establish daratumumab monotherapy efficacy or transfer to other CD38 antibodies.

Response assessment must follow the lesion. Serum free light chains and hematologic criteria measure clonal suppression, while proteinuria, estimated glomerular filtration rate, and organ-response criteria measure kidney recovery. Repeat biopsy is reserved for diagnostic uncertainty or discordant progression. In PGNMID without a detectable clone, the deposited monoclonal immunoglobulin and uncontrolled daratumumab findings support consideration of empirical plasma-cell-directed treatment, with uncertainty about the responsible clone and comparative benefit. Daratumumab-based combination therapy has an established role in AL amyloidosis; evidence in light-chain deposition disease remains observational.

### 4.4 Other and emerging indications

Daratumumab has generated observational signals in additional refractory diseases. Two patients with life-threatening anti-neutrophil cytoplasmic antibody-associated vasculitis improved after daratumumab, including pulmonary and kidney recovery, marked antibody reduction, and dialysis independence in one patient (69). A four-patient high-risk IgAN series reported a median urine protein-to-creatinine ratio change from 2.7 to 0.3 g/g, resolution of hematuria in all patients, and improvement in median estimated glomerular filtration rate during 13.5 months of follow-up (44). Individual reports also describe daratumumab for graft-versus-host disease-associated membranous nephropathy and recurrent antibody-mediated lesions (70, 71).

Across these reports, immunologic and kidney improvements were observed in selected refractory cases, but uncontrolled rescue experience cannot establish comparative benefit (44, 69–71). The clinical implications of these signals depend on response durability, the population treated, and the risks of continued or repeat therapy.

## 5 Safety, access, and clinical implementation

Implementation differs by agent and indication. Daratumumab and isatuximab have commercial supply, established pharmacy procedures, and mature hematology safety pathways, although most nonmalignant kidney uses remain off-label. Felzartamab and mezagitamab remain investigational for kidney indications. MGRS and AL amyloidosis require joint nephrology and hematology care. AMR requires a transplant protocol that integrates pathology, histocompatibility, donor-derived cell-free DNA, infection prevention, and blood-bank notification.

Finite courses, subcutaneous administration, and selective retreatment could reduce treatment burden and resource use, but formal kidney health-economic comparisons are lacking. The felzartamab AMR extension provides feasibility data for biomarker-guided dosing, without proving lower costs, better graft survival, or superiority over a fixed schedule. Choice of dose and duration remains agent-, indication-, and protocol-specific (41).

Before treatment, assessment should include complete blood count, serum immunoglobulins, hepatitis B status, vaccination and infection review, prior or active malignancy, and baseline disease-specific biomarkers. Daratumumab or isatuximab recipients require blood typing, antibody screening, and advance notification to the transfusion service because indirect antiglobulin-test interference can persist after dosing. During and after treatment, monitoring should include blood counts, immunoglobulins, infections, and disease-specific response measures, with prophylaxis and immunoglobulin replacement considered according to risk and the treatment protocol. Biomarker recurrence should prompt clinical reassessment and exclusion of alternative causes of injury; it is not an independently validated indication for anti-CD38 retreatment (34, 36, 41).

Infusion or injection reactions, infection, hypogammaglobulinemia, cytopenias, natural killer-cell depletion, transfusion-testing interference, and therapeutic monoclonal-protein interference are the principal safety and laboratory concerns. Their frequency and clinical consequences depend on the antibody, regimen, indication, and background immunosuppression (34, 36).

Infusion reactions occurred in 73% of felzartamab-treated AMR participants, but all were mild or moderate and none caused treatment discontinuation (23). In M-PLACE, 87.1% had a treatment-emergent adverse event, 29.0% had an infusion reaction, and 25.8% developed hypogammaglobulinemia (25). During isatuximab desensitization, 21.7% had grade 1–2 infusion reactions and no participant had a grade 3 or higher treatment-emergent adverse event (35). Mezagitamab treatment in IgAN produced predominantly low-grade events and no serious treatment-related events through extended follow-up (38, 39). These small, heterogeneous studies cannot establish safety during prolonged or repeated treatment, particularly in kidney-transplant recipients receiving maintenance immunosuppression (34, 36).

Prolonged or repeated natural killer-cell depletion may impair antiviral and antitumor immune surveillance. Daratumumab studies in myeloma show marked peripheral natural killer-cell depletion, but residual cells can retain cytotoxic function and cell counts alone do not establish the degree of functional impairment (72). Infection surveillance is therefore warranted during treatment and recovery. Long-term CASTOR follow-up (median 72.6 months) found more reported second primary malignancies with daratumumab-containing therapy, although exposure-adjusted rates were similar between groups (73). These findings neither establish a natural killer-cell-mediated cancer effect nor resolve risk in kidney-transplant recipients receiving triple maintenance immunosuppression. Large kidney trials with long follow-up are needed to assess malignancy and cumulative infectious risk. For nonmalignant kidney indications in patients with prior or active malignancy, anti-CD38 use remains highly experimental; whether kidney benefit outweighs tumor progression or recurrence risk is unestablished and requires individualized nephrology-oncology assessment.

The balance between response durability and cumulative risk makes disease stage and the justification for repeat treatment central to clinical interpretation.

## 6 Discussion

### 6.1 Disease stage and response durability

Pathogenic immunoglobulin production, downstream inflammation, and structural kidney damage need not resolve together. Antibody concentrations can fall before proteinuria improves, while tissue activity can change without a parallel antibody decline (18, 23, 25). Persistent proteinuria may therefore reflect continuing immune activity, delayed recovery, or residual damage; a single response measure cannot distinguish these possibilities.

M-PLACE and MONET illustrate why disease stage merits attention. M-PLACE included newly diagnosed, relapsing, and refractory pMN, whereas MONET selected patients with longstanding rituximab-resistant disease. The more limited proteinuria response in MONET suggests that prior treatment, persistent tissue disease, or residual podocyte injury may affect recovery. However, the studies differed in patient selection and follow-up and lacked concurrent controls. Their contrast cannot establish a stage-dependent treatment effect or a single resistance mechanism (25, 43).

Recurrence after finite felzartamab treatment in AMR and renewed responses after daratumumab retreatment in PGNMID show that initial response and durable control are separate outcomes (23, 30, 41). Continued antigen exposure, persistent immune deposits, or unresolved inflammation may sustain disease. Clinical relapse or antibody reappearance alone cannot identify repopulation of a particular pathogenic cell population, and these observations do not show that additional dosing can overcome resistant disease. Disease duration, prior treatment, activity, and chronic damage should be tested prospectively as selection variables; no prescribing rule based on disease stage has been validated in the reviewed studies.

### 6.2 Response assessment and biomarker-guided retreatment

Serial assessment should distinguish measured target engagement (cell counts or pharmacodynamic measures), immunologic response (pathogenic antibody or immunoglobulin), histological response (biopsy lesions), molecular response (rejection-associated transcripts), injury-biomarker response (donor-derived cell-free DNA), kidney response (proteinuria or kidney function), and hard clinical outcomes (kidney failure, graft loss, or death). A change in one domain does not establish a response in the others.

The discordance between donor-specific antibody and biopsy responses in AMR, and the lag between anti-PLA2R decline and proteinuria improvement in pMN, illustrate this distinction (18, 23, 25). These patterns make serial assessment informative but do not establish a treatment decision rule. A marker can track activity without having a validated threshold at which redosing improves clinical outcomes.

The felzartamab AMR extension tested donor-derived cell-free DNA-guided dosing after an initial fixed course. The study demonstrated feasibility, but its uncontrolled design cannot establish the benefit of biomarker-guided dosing. Persistent or recurrent AMR despite low donor-derived cell-free DNA also limits reliance on that marker alone (41). Trials comparing prespecified biomarker-guided and fixed strategies are needed to test measurement timing, thresholds, dosing decisions, organ outcomes, and cumulative harms. Monitoring a response and improving outcomes by acting on a biomarker remain distinct clinical questions.

### 6.3 Limitations and research priorities

This review used a focused narrative search without a registered systematic-review protocol. The original evidence cutoff was 17 August 2026; a dated reproducibility search and targeted checks were added during revision. Although bibliographic metadata were checked against authoritative databases, duplicate independent screening, formal risk-of-bias assessment, GRADE assessment, and quantitative synthesis were not performed. The resulting evidence map may therefore remain vulnerable to selection and interpretation bias.

The clinical literature is also heterogeneous. Indications, disease stage, prior treatment, dosing schedules, co-interventions, kidney endpoints, biomarker definitions, and follow-up differ markedly across studies. Except for AL amyloidosis, late AMR, and felzartamab-treated IgAN, most evidence comes from small uncontrolled studies, registries, or case reports. Proteinuria, donor-derived cell-free DNA, molecular biopsy scores, autoantibody concentrations, and hematologic response are clinically informative but remain surrogate or intermediate outcomes when long-term kidney survival has not been demonstrated.

Publication and sponsorship effects require particular attention. Positive rescue cases are more likely to be reported than treatment failures, conference abstracts provide incomplete methods, and several development programs are industry funded. Direct comparisons among CD38 antibodies, against contemporary combination therapy, or between biomarker-guided and fixed schedules are unavailable. Formal health-economic studies in kidney populations are also lacking. These gaps limit certainty about comparative effectiveness, optimal duration, cumulative infection risk, immunoglobulin recovery, retreatment thresholds, and cost-effectiveness.

These gaps call for trials with sufficient follow-up to assess kidney failure, sustained kidney-function loss, and graft outcomes alongside intermediate responses. Prospective analyses should test whether disease duration, activity, prior treatment, and chronic structural damage identify patients most likely to benefit. Direct comparisons should evaluate specific antibodies, finite versus continued treatment, and other treatment strategies without inferring superiority from cross-study response rates. Biomarker-guided retreatment requires prespecified measurements, timing, thresholds, and actions tested against fixed strategies. Repeated exposure also requires longer follow-up for infections, immunoglobulin recovery, and other cumulative harms.

### 6.4 Conclusions

Among the indications reviewed, the most mature randomized evidence with kidney-organ outcomes comes from daratumumab-based combination therapy in AL amyloidosis, a plasma-cell disorder (32, 33). Evidence in other kidney and transplant indications includes early-phase randomized studies, but long-term kidney or graft benefit remains unproven. Treatment effects apply to the studied antibody, regimen, population, and endpoint; the AL results do not establish daratumumab monotherapy efficacy or a class effect.

Across indications, the clinical question is whether control of pathogenic immunoglobulin production or downstream immune injury translates into durable organ benefit. Disease stage may help explain response differences, but validated selection rules are lacking. Biomarkers support monitoring, while marker recurrence alone does not justify a general retreatment rule. Limited comparative evidence and uncertainty about cumulative harm constrain use outside established indications. Research should prioritize kidney and graft outcomes, patient selection by disease stage, comparisons of agents and treatment strategies, validation of biomarker-guided retreatment, and safety during repeated treatment.
