# Supplementary material for: CD38-targeted therapies in kidney disease: evidence maturity, disease-stage dependence, and biomarker-guided retreatment
Source: Front Med (Lausanne). 2026 Oct 14;13:1972506. doi: 10.3389/fmed.2026.1972506 (PMC13714534; doi:10.3389/fmed.2026.1972506)
Supplement: Supplementary file 1 [file Supplementaryfile1.docx]

Supplementary Methods S1 Search documentation

CD38-Targeted Therapies in Kidney Disease: Evidence Maturity, Disease-Stage Dependence, and Biomarker-Guided Retreatment

# Scope and dates

This focused narrative review retains the original literature cutoff of 17 August 2026. To make the retrieval reproducible, the clinical and mechanistic queries below were specified and executed on 10 September 2026 using that cutoff. These are revision-stage search records. The historical query log was not available for retrospective reconstruction, so the counts below are not presented as the original search yield or as a systematic-review screening flow.

Database: PubMed, including MEDLINE-indexed records and other records available through PubMed. Interface: NCBI E-utilities ESearch, followed by EFetch for record retrieval. Date field: publication date (pdat). The explicit API bounds were 1800/01/01 to 2026/08/17, equivalent to no substantive lower date limit for PubMed. No language or publication-type filters were applied, and no human-only filter was imposed at retrieval. Clinical relevance and original human evidence were considered during narrative selection.

# Complete clinical query

("CD38"[Title/Abstract] OR "CD38 Antigens"[MeSH Terms] OR "anti-CD38"[Title/Abstract] OR daratumumab[Title/Abstract] OR felzartamab[Title/Abstract] OR isatuximab[Title/Abstract] OR mezagitamab[Title/Abstract] OR MOR202[Title/Abstract] OR BIIB148[Title/Abstract] OR "TAK-079"[Title/Abstract]) AND (kidney[Title/Abstract] OR renal[Title/Abstract] OR nephropathy[Title/Abstract] OR nephritis[Title/Abstract] OR glomerulonephritis[Title/Abstract] OR "Kidney Diseases"[MeSH Terms] OR "Kidney Transplantation"[MeSH Terms] OR "antibody-mediated rejection"[Title/Abstract] OR desensitization[Title/Abstract] OR "IgA nephropathy"[Title/Abstract] OR "membranous nephropathy"[Title/Abstract] OR "lupus nephritis"[Title/Abstract] OR "ANCA-associated vasculitis"[Title/Abstract] OR "monoclonal gammopathy of renal significance"[Title/Abstract] OR PGNMID[Title/Abstract] OR "light chain deposition disease"[Title/Abstract] OR "light-chain amyloidosis"[Title/Abstract] OR "AL amyloidosis"[Title/Abstract])

# Complete mechanistic query

("CD38"[Title/Abstract] OR "CD38 Antigens"[MeSH Terms] OR "anti-CD38"[Title/Abstract] OR daratumumab[Title/Abstract] OR felzartamab[Title/Abstract] OR isatuximab[Title/Abstract] OR mezagitamab[Title/Abstract] OR MOR202[Title/Abstract] OR BIIB148[Title/Abstract] OR "TAK-079"[Title/Abstract]) AND (("plasma cells"[Title/Abstract] OR plasmablast*[Title/Abstract] OR "natural killer"[Title/Abstract] OR "NK cells"[Title/Abstract]) AND (kidney[Title/Abstract] OR renal[Title/Abstract] OR transplant*[Title/Abstract]))

# Retrieval counts and interpretation

| **Retrieval component** | **Records** |
| --- | --- |
| Clinical query | 958 |
| Mechanistic query | 386 |
| Overlap by PMID | 202 |
| Unique retrieved PMIDs | 1142 |

The unique count was obtained by taking the union of the two PMID lists. DOI metadata were also checked for record identity. These counts describe retrieval only; they do not assert that every retrieved record underwent a systematic eligibility assessment. Tables 3 and 4 contain 13 selected clinical evidence entries, with follow-up reports and overlapping cohorts identified as such. An evidence entry is not necessarily an independent study or a unique patient cohort.

# Source selection and publication status

Original human reports with kidney, immunologic, histological, molecular, or transplant-access outcomes were prioritized for clinical claims. Mechanistic studies, reviews, and guidelines supply biological or clinical context. Studies confined to hematologic malignancy without a kidney or mechanistic contribution are outside the kidney-efficacy synthesis. The reviewer-requested CASTOR and natural killer-cell studies provide indirect safety and mechanistic context; they are not kidney-efficacy evidence.

Conference abstracts and preprints are treated as preliminary reports when no full peer-reviewed report is available. Their stated design and publication status are recorded separately; incomplete reporting limits appraisal. A later full report supersedes an abstract for shared endpoints. Trial registrations without peer-reviewed efficacy results are listed separately in the development table. No preprint-only clinical entry is used in Tables 3 or 4.

# Duplicate and overlapping reports

Database records were deduplicated by PMID, with DOI used to check identity. Clinical reports were linked using trial identifiers, authors, recruitment setting, sample characteristics, and schedules where available. A later report was preferred for the shared endpoint when it supplied more complete or longer follow-up; an earlier report or extension was retained when it supplied distinct mechanistic, endpoint, or retreatment information. Participants from linked reports were not treated as independent replication.

| **Report family** | **Handling in this review** |
| --- | --- |
| Felzartamab AMR trial and extension (18, 23, 41, 49) | The randomized trial and selected 11-patient extension are shown separately for their different treatment questions. Molecular and prognostic analyses are linked to the parent trial. The extension is not independent randomized replication. |
| IGNAZ (24, 42) | The publication and registry describe the same study. The 48 randomized participants and the six Japanese open-label participants are distinguished, as are 24- and 12-month follow-up. |
| Mezagitamab IgAN (37–39) | The registry and week-48/week-96 conference reports are treated as one development cohort, not independent efficacy studies. Findings remain preliminary. |
| ANDROMEDA (32, 33) | The initial and final follow-up reports concern the same randomized trial. The initial report supplies kidney-organ response; final follow-up supplies longer-term outcomes. |
| Other observational reports | Distinct reports are not assumed to contain unique patients when overlap cannot be excluded. No pooled patient total or meta-analysis is presented. |

# Targeted revision checks

The revision also checked the original reports and official sources relevant to the reviewer comments, including AMR molecular findings, the biomarker-guided extension, M-PLACE, MONET, IGNAZ, the Banff 2022 definitions, and the specified long-term daratumumab safety report.

ClinicalTrials.gov records NCT07219043 and NCT07444489 were checked on 10 September 2026. Their official titles, design, planned enrollment, status, principal outcomes, and completion dates were entered in Table 5, with the access date stated separately from the original five entries. The new records had no posted results in the retrieved registry data. Registration alone provides no clinical efficacy evidence.

# Search limitations

The search is focused and non-systematic. There was no registered review protocol, duplicate independent screening, formal risk-of-bias assessment, or quantitative synthesis. Broad retrieval terms do not guarantee comprehensive inclusion. The documented revision-stage queries improve reproducibility of retrieval while the selective narrative design remains a limitation.
